# Effect of Non-selective Beta-Blockers on the Prevention of Decompensation in Compensated Cirrhosis: A Systematic Review

**DOI:** 10.7759/cureus.103468

**Published:** 2026-02-12

**Authors:** Ishtiaq Ahmad, Shivam Singla, Ali Nasir, Bhavna Singla, Ajay, Sunita Kumawat, Sajid Abbas

**Affiliations:** 1 Internal Medicine, University of Iowa Hospitals and Clinics, Iowa City, USA; 2 Internal Medicine, TidalHealth Peninsula Regional, Salisbury, USA; 3 Gastroenterology and Hepatology, Pakistan Kidney and Liver Institute and Research Centre, Lahore, PAK; 4 Internal Medicine, Erie County Medical Center, Buffalo, USA; 5 Internal Medicine, Liaquat University of Medical and Health Sciences, Jamshoro, PAK; 6 Internal Medicine, St. Francis Medical Center, Lynwood, USA; 7 Internal Medicine, Holy Family Hospital, Rawalpindi, PAK

**Keywords:** carvedilol, clinically significant portal hypertension, compensated cirrhosis, decompensation, non-selective beta-blockers, portal hypertension management, propranolol, variceal progression

## Abstract

This systematic review evaluates the effectiveness of non-selective beta-blockers (NSBBs) in preventing first decompensation and disease progression in patients with compensated cirrhosis, with emphasis on those with clinically significant portal hypertension (CSPH). Six randomized controlled trials (RCTs) were analyzed, representing both historic endoscopy-based studies and modern hemodynamically defined cohorts. The synthesis demonstrates that NSBBs confer meaningful benefit only after the hemodynamic threshold of CSPH is reached, as exemplified by contemporary trials showing reduced risk of decompensation, particularly ascites, while earlier studies enrolling patients without confirmed CSPH or with mild portal hypertension did not demonstrate benefit and, in some cases, suggested potential harm. Carvedilol showed additional promise in delaying variceal progression even with modest HVPG reduction, indicating potential mechanisms beyond portal pressure lowering. Across trials, the variability in outcomes was explained by differences in baseline hemodynamic severity, selection criteria, and methodological rigor, highlighting the importance of disease-stage-specific application. Overall, the findings support a shift toward targeted NSBB therapy in compensated cirrhosis with confirmed or probable CSPH, rather than universal prophylaxis in all compensated patients.

## Introduction and background

Portal hypertension is the central pathophysiological hallmark of cirrhosis and the primary determinant of clinical progression from a compensated to a decompensated state. The development of clinically significant portal hypertension (CSPH), defined as a hepatic venous pressure gradient (HVPG) of ≥10 mmHg, marks a critical transition point at which the risk of first decompensation - ascites, variceal bleeding, or hepatic encephalopathy - increases substantially [1,2]. Patients may remain clinically compensated for years; however, once decompensation occurs, prognosis declines sharply, with a reported reduction in median survival from more than 12 years in compensated cirrhosis to fewer than two years after the onset of complications. Preventing this transition has therefore become a major focus of modern hepatology, especially as the field increasingly recognizes cirrhosis as a dynamic, treatable disease rather than an irreversible end stage of liver injury [3].

Non-selective beta-blockers (NSBBs), including propranolol, nadolol, and carvedilol, have long been established as first-line therapy for preventing variceal hemorrhage through their dual mechanism of reducing portal inflow (via β1 blockade decreasing cardiac output) and splanchnic vasoconstriction (via β2 blockade unopposed α-adrenergic activity) [4]. Carvedilol additionally offers intrinsic anti-α1 activity, resulting in a greater reduction in intrahepatic resistance and a more pronounced decline in portal pressure. Historically, NSBBs were introduced for the prevention of variceal bleeding in patients with medium or large varices. However, emerging evidence over the past decade has expanded their role considerably [5]: NSBBs may provide broader disease-modifying benefits by lowering portal pressure early in the course of cirrhosis, thereby delaying or preventing the onset of decompensation. This shift is supported by the pivotal recognition that portal hypertension itself, not variceal bleeding alone, is the driving force behind clinical deterioration [6].

Recent randomized controlled trials (RCTs), particularly in patients with compensated cirrhosis and CSPH, have demonstrated that NSBBs can significantly reduce the incidence of first decompensation, driven predominantly by a reduction in ascites formation. These findings have influenced modern clinical guidelines, reinforcing the concept that early pharmacologic reduction of portal pressure may alter the natural history of cirrhosis [7,8]. Nevertheless, evidence remains heterogeneous with respect to patient selection, drug class, dosing strategies, and long-term survival outcomes. As such, questions persist regarding the strength and consistency of NSBB benefits across different compensated populations, especially those with no or small varices. The objective of this systematic review is to critically evaluate and synthesize evidence from RCTs assessing the effectiveness of NSBBs in preventing first decompensation and reducing mortality in patients with compensated cirrhosis and CSPH.

## Review

Materials and methods

Study Design and Reporting Framework

This systematic review was conducted in accordance with the Preferred Reporting Items for Systematic Reviews and Meta-Analyses (PRISMA) guidelines [9]. All methodological steps, including search strategy development, study selection, data extraction, and risk of bias assessment, were performed following a predefined protocol designed to ensure transparency and reproducibility. Because the aim of this review was to evaluate the highest level of evidence regarding the effect of NSBBs on preventing decompensation in compensated cirrhosis with CSPH, only RCTs were included. This decision was made to minimize confounding inherent in observational studies and to ensure that any conclusions regarding causality are grounded in the most robust trial data available.

PICO Framework and Eligibility Criteria

The review question was structured using the Population, Intervention, Comparator, and Outcome (PICO) format [10]. The population consisted of adults with compensated cirrhosis, with or without the presence of small esophageal varices, and with or without direct hemodynamic confirmation of CSPH, depending on the study methodology. The intervention of interest was any NSBB, including propranolol, nadolol, timolol, or carvedilol, administered for the primary purpose of reducing portal pressure or preventing early clinical decompensation. Comparator groups included placebo or no active beta-blocker therapy. Eligible studies were required to evaluate at least one clinically relevant outcome related to hepatic decompensation, progression of portal hypertension, development or progression of esophageal varices, variceal bleeding, or all-cause mortality. Only RCTs published in peer-reviewed journals were included, with no restrictions on publication year. Observational studies, prospective cohorts without randomization, non-randomized hemodynamic trials, conference abstracts, narrative reviews, and studies exclusively assessing procedural or non-pharmacologic interventions were excluded to maintain internal validity and preserve the focus on causative inference.

Information Sources and Search Strategy

A comprehensive literature search was performed across PubMed/MEDLINE, Embase, and the Cochrane Central Register of Controlled Trials (CENTRAL), covering all records from database inception to December 31, 2023. Additional sources included manual screening of reference lists of relevant articles and prior systematic reviews. The search strategy combined Medical Subject Headings (MeSH) and free-text terms using Boolean operators, including expressions such as “Portal Hypertension”[MeSH] AND “Cirrhosis”[MeSH], “beta-blocker” OR “propranolol” OR “nadolol” OR “carvedilol” OR “timolol”, and outcome-related terms such as “ascites,” “variceal bleeding,” “decompensation,” and “Hepatic Encephalopathy”[MeSH]. Randomized trial filters were applied using terms such as “Randomized Controlled Trial”[Publication Type] OR “random*” in combination with disease-specific terminology. Search terms were adapted for each database to account for differences in indexing and syntax. No language or date restrictions were applied, and the final search was completed on December 31, 2023.

Study Selection Process

Titles and abstracts retrieved from the search were independently screened by reviewers to identify potentially relevant studies. Full-text articles were then evaluated for eligibility based on the predefined inclusion and exclusion criteria. Any disagreements regarding study inclusion were resolved through discussion and consensus. This process ensured adherence to PRISMA methodology while minimizing the risk of selection bias. RCTs investigating NSBBs for the prevention of decompensation, variceal progression, or variceal bleeding in compensated cirrhosis were included, even when CSPH was defined variably (endoscopic surrogates vs direct HVPG measurement). This decision was justified by the need to capture the full spectrum of early compensated disease and reflects the historical evolution of how CSPH has been operationalized in clinical research. We acknowledge that the inclusion of trials defining CSPH using heterogeneous criteria, ranging from endoscopic surrogates (e.g., variceal size) to direct HVPG measurement, introduces pathophysiologic heterogeneity across studies. This variability likely contributes to differences in observed treatment effects and was therefore considered when interpreting comparative efficacy in the synthesis.

Data Extraction and Management

Data extraction was performed using a structured table designed to capture all key study characteristics, including study design, sample size, population features, type and dosing of NSBB used, comparator details, duration of follow-up, outcomes assessed, main effect estimates, and adverse events. The extraction approach ensured consistent and comprehensive documentation of trial-level data. Because the included studies varied in outcome definitions and follow-up durations, extraction focused on clinically comparable endpoints such as variceal progression, first decompensation, variceal bleeding, and mortality, which would later be described in the Results section. Extracted data were cross-verified for accuracy.

Risk-of-Bias Assessment

Each included RCT underwent methodological appraisal using the Cochrane Risk of Bias 2 (RoB 2) tool [11]. Domains assessed included the randomization process, deviations from intended interventions, completeness of outcome data, measurement of outcomes, and selective reporting. Judgments were categorized as low risk, some concerns, or high risk.

Synthesis Approach

A meta-analysis was not performed due to substantial heterogeneity across trials. Included RCTs differed in key design elements, including the definition of CSPH (direct HVPG measurement versus endoscopic surrogates), primary endpoints (variceal progression, first decompensation, variceal bleeding, mortality), duration of follow-up, and type and titration strategy of NSBB used. These methodological and clinical differences resulted in non-comparable effect measures, making quantitative pooling inappropriate and potentially misleading. Although subgroup pooling was considered, only one trial exclusively enrolled patients with HVPG-confirmed CSPH, precluding meaningful meta-analytic synthesis even within this subgroup. For these reasons, a structured narrative synthesis was undertaken in accordance with PRISMA and Cochrane methodological guidance.

Results

Study Selection Process

**Figure 1:**
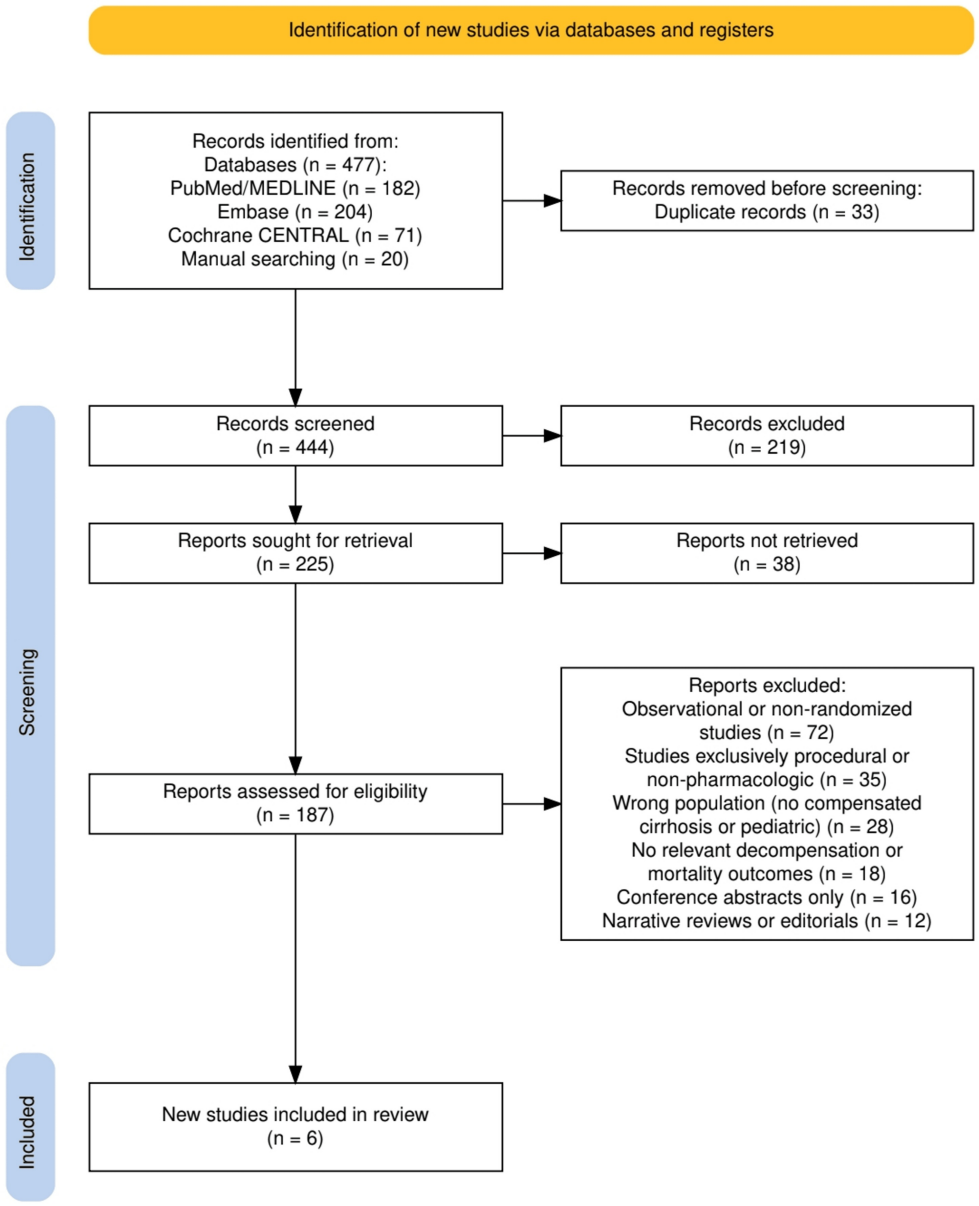
The PRISMA flowchart represents the study selection process. PRISMA: Preferred Reporting Items for Systematic Reviews and Meta-Analyses

The study selection process is outlined in Figure 1, which details the flow of records from initial identification through final inclusion. A total of 477 records were retrieved across all databases, after which 33 duplicates were removed, leaving 444 records for title and abstract screening. Of these, 219 were excluded for not meeting eligibility criteria based on the predefined PICO framework. Full-text assessment was conducted for 187 reports, from which 181 were excluded for reasons specified in Figure 1, including observational or otherwise non-randomized designs, studies limited to procedural or non-pharmacologic interventions, populations outside compensated cirrhosis, absence of relevant clinical outcomes, conference abstracts without full reports, and narrative reviews or editorials. Ultimately, six RCTs met all inclusion criteria and were incorporated into the final synthesis, forming the evidence base for evaluating the role of NSBBs in preventing decompensation in compensated cirrhosis with CSPH.

Characteristics of the Selected Studies

The characteristics of the selected RCTs are summarized in Table 1 and reflect the evolution of research on NSBBs in compensated cirrhosis over the past three decades. The six included trials varied substantially in population profiles, hemodynamic definitions, and therapeutic strategies, providing a broad view of how NSBBs have been applied across different stages of portal hypertension. Early studies, such as those by Calès and Groszmann, predominantly enrolled compensated patients without confirmed CSPH and relied on endoscopic markers or minimal HVPG thresholds, whereas more contemporary trials, particularly PREDESCI [12], exclusively included patients with HVPG ≥10 mmHg and stratified therapy based on hemodynamic responsiveness. Interventions also differed, with propranolol, nadolol, timolol, and carvedilol used at variable doses and titration protocols tailored to heart rate reduction or hemodynamic response. Follow-up durations ranged from two to five years, and outcomes focused on variceal progression, first decompensation events, and mortality. Collectively, these trials provide a heterogeneous yet informative evidence base that allows for meaningful comparison of NSBB efficacy across different clinical and hemodynamic contexts in compensated cirrhosis.

**Table 1:** Summary of randomized controlled trials evaluating non-selective beta-blockers for the prevention of decompensation and variceal progression in compensated cirrhosis. NSBB: non-selective beta-blocker, CSPH: clinically significant portal hypertension, HVPG: hepatic venous pressure gradient, RCT: randomized controlled trial, HE: hepatic encephalopathy, HR: hazard ratio, CI: confidence interval, NAFLD: non-alcoholic fatty liver disease

Study ID (author, year)	Study design	Sample size (total / per arm)	Population characteristics	NSBB intervention (drug + dose/titration)	Comparator	Follow-up duration	Outcome(s) measured	Key findings / effect estimates	Adverse events
Villanueva et al., 2019 (PREDESCI) [12]	Randomised, double-blind, placebo-controlled, multicentre RCT	N = 201 (NSBB = 100; Placebo = 101)	Compensated cirrhosis + CSPH (HVPG ≥10 mmHg), no high-risk varices; HVPG measured in all patients classified as acute responders vs non-responders	Responders → Propranolol up to 160 mg BID; Non-responders → Carvedilol ≤25 mg/day. Dose is individually titrated during the open-label phase, then randomized	Placebo	Not explicitly stated in the abstract; trial recruitment 2010–2013, long-term follow-up until endpoint occurrence	Primary: Decompensation (ascites, bleeding, overt HE) or death	Primary endpoint: 16% (NSBB) vs. 27% (placebo); HR 0.51 (95% CI 0.26–0.97), p = 0.041. Major effect driven by ↓ ascites: HR 0.44, p = 0.0297	Overall adverse events similar between groups; six severe AEs (4 in the NSBB group)
Groszmann et al., 2005 (NEJM) [13]	Randomised, double-blind, placebo-controlled RCT	N = 213 (Timolol = 108; Placebo = 105)	Cirrhosis with portal hypertension; baseline HVPG ≥ 6 mmHg; no varices at baseline; compensated disease	Timolol, non-selective β-blocker; oral dose titrated to HR reduction per protocol (exact titration details in full text)	Placebo	Median follow-up: 54.9 months	Primary: Development of gastroesophageal varices OR variceal hemorrhage. Secondary: Ascites, encephalopathy, transplant, death	No difference in primary endpoint: 39% (timolol) vs 40% (placebo), P = 0.89. No significant differences in ascites, encephalopathy, transplant, or mortality. HVPG responders (<10 mmHg or ↑ >10%) showed differing variceal progression rates	Serious AEs: 18% in timolol vs 6% placebo (P = 0.006). Higher frequency of adverse events in the NSBB group
Merkel et al., 2004 [14]	Randomised, placebo-controlled clinical trial	N = 161 (nadolol = 83; placebo = 78)	Compensated cirrhosis with small esophageal varices (F1); no previous bleeding; baseline characteristics balanced	Nadolol, mean dose 62 ± 25 mg/day, titrated to achieve 25% reduction in resting HR	Placebo	Mean follow-up: 36 months (endoscopy at 12, 24, 36, 48, 60 months)	Primary: Growth of varices (F1 → F2/F3). Secondary: Variceal bleeding, survival	Growth of varices: 9 (nadolol) vs 29 (placebo); cumulative risk 20% vs 51%, P < 0.001. OR for progression: 4.0 (95% CI 1.95–8.4). Variceal bleeding also reduced (P = 0.02). Survival was not different (P = 0.33)	Withdrawal due to AEs: nine in nadolol vs. one in placebo (P = 0.01)
Sarin et al., 2013 [15]	Randomised, placebo-controlled clinical trial; 3 × 2 factorial design (NSBB vs. placebo; HVPG strategies)	N = 150 (Propranolol = 77; Placebo = 73)	Compensated cirrhosis; small esophageal varices (≤5 mm); no previous variceal bleeding; etiologies mainly viral and alcohol	Propranolol, titrated to achieve target HR reduction (exact dose individualized; typical NSBB titration protocol used)	Placebo	2-year follow-up for primary endpoint	Primary: Growth of varices. Secondary: Variceal bleeding, mortality, effect of HVPG monitoring strategy	2-year actuarial variceal growth: 11% (propranolol) vs 16% (placebo), P = 0.786 → no significant effect. Variceal bleeding and mortality are also not different. HVPG measurement strategy did not influence outcomes. Bilirubin ≥1.5 mg/dL predicted variceal progression	No major difference reported between groups
Bhardwaj et al., 2017 [16]	Randomised, placebo-controlled trial	N = 140 (Carvedilol = 70; Placebo = 70)	Compensated cirrhosis with small oesophageal varices; predominant etiology NAFLD; baseline characteristics comparable	Carvedilol, mean dose 12 ± 1.67 mg/day, titrated to target HR (58 ± 3 bpm)	Placebo	Minimum 24 months; endoscopy every 6 months; HVPG measured at baseline and 12 months	Primary: Progression from small to large varices. Secondary: HVPG change, mortality, variceal bleeding	Non-progression to large varices: 79.4% (carvedilol) vs 61.4% (placebo); p = 0.04. Mean time to non-progression: 20.8 months vs 18.7 months (p = 0.04). HVPG reduction modest: –8.64% vs +0.33% (p = 0.22). No variceal bleeding deaths or liver-related deaths in either group	No major adverse events reported in either group
Calès et al., 1999 [17]	Randomised, double-blind, placebo-controlled clinical trial	N = 206 (Propranolol = 102; Placebo = 104)	Cirrhosis with no varices or small varices at baseline; compensated stage; clinical/biochemical characteristics similar between groups	Long-acting propranolol 160 mg/day (fixed dose regimen)	Placebo	Two years (video-recorded endoscopic assessment)	Primary: Development of large varices. Secondary: Variceal bleeding, mortality	At two years, progression to large varices: 31% (propranolol) vs 14% (placebo) (P < 0.05) → propranolol did NOT prevent progression. Bleeding: 3 vs 4 cases. Mortality: 9 vs 10 deaths → no significant difference	~1/3 lost to follow-up; compliance high among retained participants

Risk-of-Bias Assessment

The risk-of-bias assessment for the included RCTs, summarized in Table 2, revealed variation in methodological quality across the evidence base. More recent trials demonstrated an overall low risk of bias due to robust randomization procedures, blinding, and clearly defined clinical outcomes, whereas older studies showed a higher likelihood of bias related to incomplete reporting of methodology, loss to follow-up, and less standardized assessment of portal hypertension. Several trials were graded as having some concerns, primarily due to limited detail on allocation concealment or attrition handling, although outcome measurements were generally reliable across studies. These differences in methodological rigor help contextualize variability in treatment effects and reinforce the need to interpret earlier findings with appropriate caution.

**Table 2:** Risk-of-bias assessment of randomized controlled trials evaluating non-selective beta-blockers in compensated cirrhosis. RoB 2: Cochrane Risk of Bias 2 tool, RCT: randomized controlled trial, HVPG: hepatic venous pressure gradient

Study ID	Randomization process	Deviations from intended interventions	Missing outcome data	Outcome measurement	Selective reporting	Overall RoB
Villanueva et al., 2019 (PREDESCI) [12]	Low risk – central randomization, double-blind	Low risk – well-controlled, placebo-blinded	Low risk – minimal loss, ITT used	Low risk – objective clinical outcomes	Low risk – pre-registered, outcomes reported	Low risk
Groszmann et al., 2005 (NEJM) [13]	Low risk – robust RCT methods	Low risk – double-blind, good adherence	Low risk – losses small, balanced	Low risk – endoscopy/HVPG standardized	Low risk – protocolized reporting	Low risk
Merkel et al., 2004 [14]	Some concerns – randomization details limited	Low risk – placebo-controlled	Some concerns – moderate withdrawals	Low risk – scheduled endoscopies	Some concerns – no protocol available	Some concerns
Sarin et al., 2013 [15]	Some concerns – limited description of randomization	Low risk – good intervention control	Some concerns – attrition not fully detailed	Low risk – objective endpoints	Some concerns – unclear protocol	Some concerns
Bhardwaj et al., 2017 [16]	Some concerns – randomization details sparse	Low risk – blinded and protocolized dosing	Low risk – minimal dropout	Low risk – objective/endoscopic assessment	Some concerns – limited protocol detail	Some concerns
Calès et al., 1999 [17]	Some concerns – older RCT with less methodological detail	Low risk – blinded propranolol vs placebo	High risk – ~1/3 lost to follow-up	Low risk – endoscopy-based measurement	Some concerns – pre-registration absent	High risk

Discussion

This systematic review demonstrates that the effectiveness of NSBBs in compensated cirrhosis depends fundamentally on the underlying severity of portal hypertension and the clinical stage at which treatment is initiated. Across six RCTs, a clear divergence emerges between modern hemodynamically defined CSPH populations and earlier RCTs that relied solely on endoscopic surrogates such as the presence of small varices. The PREDESCI trial [12], the only study to exclusively enroll patients with confirmed CSPH (HVPG ≥10 mmHg), showed a significant reduction in the composite endpoint of first decompensation or death, with a hazard ratio of 0.51 and a particularly notable reduction in new-onset ascites. This contrasts sharply with earlier trials such as the NEJM timolol study, which included patients with only mild portal hypertension (HVPG ≥6 mmHg) and no varices; in this lower-risk population, NSBBs provided no prophylactic benefit and were accompanied by a higher incidence of adverse events. Similarly, propranolol trials in patients with small varices produced heterogeneous findings, with Merkel et al. [14] demonstrating delayed variceal progression with nadolol, whereas Sarin et al. [15] reported no benefit of propranolol in preventing variceal growth or bleeding. The carvedilol trial by Bhardwaj et al. [16] adds further nuance, showing a favorable effect on variceal progression despite only modest HVPG reductions, suggesting that carvedilol’s combined β-blockade and α1-antagonism might confer additional microvascular or anti-fibrotic benefits beyond simple portal pressure reduction. Meanwhile, the older Calès study [17] not only failed to show benefit but paradoxically observed greater variceal progression in the propranolol arm, an effect likely influenced by substantial loss to follow-up and the inclusion of patients without CSPH.

Taken together, these findings align with the contemporary understanding that NSBBs exert their disease-modifying effects primarily in patients who have transitioned from subclinical portal hypertension to CSPH, a stage at which sinusoidal resistance, splanchnic vasodilation, and portal inflow abnormalities create a hemodynamic environment amenable to therapeutic modulation [18]. The contrasting results across trials underscore that NSBBs are not universally effective in all compensated patients but rather exert maximal benefit in those with demonstrable portal pressure thresholds and early pathophysiologic vulnerability to decompensation. In this context, the collective RCT evidence reinforces a shift away from the earlier universal prophylactic model toward a more targeted strategy consistent with current Baveno and AASLD guidance, which positions NSBBs as a first-line therapy specifically for patients with CSPH, independent of variceal size [19].

The biological foundation for using NSBBs early in compensated cirrhosis lies in their ability to modulate the core hemodynamic disturbances responsible for the transition to decompensation. NSBBs reduce portal pressure through a dual mechanism: β1-blockade decreases cardiac output and portal venous inflow, while β2-blockade promotes unopposed splanchnic vasoconstriction that limits hyperdynamic circulation and splanchnic pooling-key drivers of portal hypertension [20]. Carvedilol further augments this effect through mild α1-adrenergic antagonism, lowering intrahepatic resistance and thereby achieving greater reductions in HVPG. Because CSPH (HVPG ≥10 mmHg) marks the threshold at which ascites, variceal bleeding, and encephalopathy emerge, therapies that can stabilize or reverse these pressure gradients have the potential to delay decompensation [21]. Increasing evidence also suggests that NSBBs mitigate systemic inflammation, impair bacterial translocation, and reduce sympathetic overactivation, all of which contribute to microvascular dysfunction and progression of portal hypertension. These mechanistic pathways provide a compelling physiologic rationale for initiating NSBB therapy before overt complications arise.

The conflicting results between earlier RCTs and more contemporary evidence reflect differences in patient selection, disease stage, and the precision of portal hypertension assessment. Early trials, such as the NEJM timolol study and propranolol studies in small varices, enrolled broad compensated populations without requiring CSPH confirmation. Many participants had only mild portal hypertension (HVPG between 6 and 9 mmHg), a stage where the biological substrate necessary for NSBB benefit, marked splanchnic vasodilation, and elevated intrahepatic resistance, may not yet be present [22,23]. Moreover, reliance on endoscopic surrogates to represent portal hypertension likely introduced misclassification bias, as variceal size does not uniformly correlate with hemodynamic severity. In contrast, PREDESCI restricted enrollment to patients with HVPG ≥10 mmHg, a pathophysiologic threshold firmly associated with future decompensation, and titrated therapy based on individual hemodynamic response [12]. This more refined selection produced a clear therapeutic signal absent in earlier heterogeneous cohorts. The divergence highlights a fundamental concept: NSBB efficacy is not uniform across the compensated spectrum but is contingent upon the underlying hemodynamic milieu. Modern evidence implies that NSBBs should be viewed as disease-modifying agents for patients with established CSPH rather than broad prophylactics for all compensated cirrhotics.

The variability in treatment effects across RCTs underscores the importance of identifying which compensated patients are most likely to derive meaningful benefit from NSBB therapy. While HVPG measurement remains the gold standard for confirming CSPH (HVPG ≥10 mmHg), we recognize that many centers lack access to hemodynamic studies. In such settings, validated non-invasive markers serve as practical surrogates for identifying patients with probable CSPH. These include liver stiffness measurements combined with thrombocytopenia, as incorporated in the Baveno VI and VII criteria, where liver stiffness >20-25 kPa together with platelet counts <150,000/mm³ reliably predicts CSPH and the presence of high-risk portal hypertension. Additionally, splenomegaly, though variably reported in clinical trials, correlates with portal hypertension severity and provides a readily available marker in resource-limited environments [24,25]. Patients with small varices and supplementary risk factors-such as elevated bilirubin, rapid variceal progression, or metabolic/steatosis-driven portal hypertension-may also benefit, particularly when carvedilol is used. Conversely, individuals without CSPH or those with minimal portal hypertension appear unlikely to respond, as supported by early RCTs demonstrating no reduction in downstream clinical events in this lower-risk group. Collectively, these insights support an individualized, pathophysiology-based approach to NSBB initiation, consistent with current guideline trends that recommend therapy guided by probability of CSPH, rather than variceal size alone [26,27].

This review adds important clarity to the evolving role of NSBBs in the early stages of portal hypertension by synthesizing evidence across distinct eras of clinical trial design. By directly comparing older endoscopy-based RCTs with contemporary hemodynamically defined studies such as PREDESCI [12], the review highlights a previously underappreciated stage-specific treatment effect: NSBBs demonstrate meaningful benefit only after the threshold of CSPH has been reached. Integrating methodological insights, including differences in HVPG confirmation, attrition patterns, and hemodynamic titration strategies, the review situates each trial within its historical and scientific context and resolves apparent contradictions in the literature. This distinct synthesis not only clarifies why prior studies reported heterogeneous findings but also reframes NSBBs as disease-modifying therapies for a well-defined subgroup rather than broad prophylactics for all compensated cirrhotics. In doing so, the review advances the field by providing a more mechanistically grounded and clinically coherent interpretation of existing evidence.

Despite promising findings, several limitations within the current evidence base constrain definitive conclusions regarding the full scope of NSBB benefits in compensated cirrhosis. Mortality effects remain uncertain, with PREDESCI [12] suggesting a trend toward improved survival but older trials showing no significant impact, likely due to low event rates and insufficient power. Heterogeneity in defining CSPH across studies complicates synthesis, as earlier RCTs relied on endoscopic markers rather than direct HVPG measurement, introducing variability in disease severity and potentially diluting treatment effects. Furthermore, the absence of standardized HVPG thresholds and inconsistent titration protocols across trials limits the comparability of the hemodynamic response. Older studies, particularly Calès et al. [17], suffered from substantial attrition, raising concerns about bias and the reliability of outcome estimates. Additional gaps include the unclear role of NSBBs in non-viral etiologies such as NAFLD cirrhosis and the lack of trials assessing biomarker-driven or HVPG-guided therapy. These limitations highlight the need for contemporary, precision-designed RCTs to refine patient selection and quantify long-term clinical benefit more accurately.

Future research must adopt precision-based approaches that align therapy with the underlying pathophysiology of compensated cirrhosis rather than relying solely on variceal size or clinical staging. Trials incorporating routine HVPG measurement, or validated non-invasive surrogates such as liver stiffness and platelet-based algorithms, are needed to more accurately identify patients with true CSPH who are most likely to benefit from NSBB therapy. Comparative trials evaluating carvedilol-first strategies, given its favorable hemodynamic and microvascular effects, may help redefine first-line pharmacologic management. There remains an urgent need for studies integrating biomarkers of systemic inflammation, sympathetic overactivation, endothelial dysfunction, or portal pressure trajectories to refine risk prediction and optimize timing of therapy. Additionally, adaptive trial designs that modulate treatment according to early hemodynamic response, such as HVPG-guided dose titration, could clarify which subpopulations derive the most clinically meaningful benefit. Collectively, these directions aim to bridge the gap between mechanistic understanding and individualized clinical practice.

## Conclusions

The available randomized evidence, although limited in number and heterogeneous in methodology, supports the selective use of NSBBs in patients with compensated cirrhosis who have developed CSPH. Within this subgroup, these agents appear to reduce the risk of first decompensation, particularly ascites, whereas trials enrolling patients without CSPH or only mild portal hypertension have not demonstrated comparable benefit. These observations suggest that the therapeutic effect of NSBBs is stage-specific rather than universally applicable across all compensated patients. Given the absence of pooled quantitative estimates and the variability in CSPH assessment across studies, the certainty and generalizability of this effect should be interpreted cautiously. Nonetheless, the overall direction of evidence supports a risk-based, individualized approach in which accurate identification of CSPH-using HVPG or validated non-invasive surrogates guides the targeted deployment of NSBB therapy in patients most likely to benefit.
